# 2,4-Diiodo­aniline

**DOI:** 10.1107/S1600536809030438

**Published:** 2009-08-08

**Authors:** Graham Smith, Urs D. Wermuth

**Affiliations:** a School of Physical and Chemical Sciences, Queensland University of Technology, GPO Box 2434, Brisbane, Qld 4001, Australia

## Abstract

The structure of the title compound, C_6_H_5_I_2_N, shows a weak inter­molecular amine–amine N—H⋯N hydrogen-bonding inter­action, giving a helical chain which extends along the *a* axis. An intra­molecular N—H⋯I hydrogen bond is also observed.

## Related literature

For related structures, see: Garden *et al.* (2002 ▶). For the synthesis, see: Dains *et al.* (1935 ▶); Hodgson & Marsden (1937 ▶); O’Neil (2001 ▶). For graph-set analysis of hydrogen bonding, see: Etter *et al.* (1990 ▶).
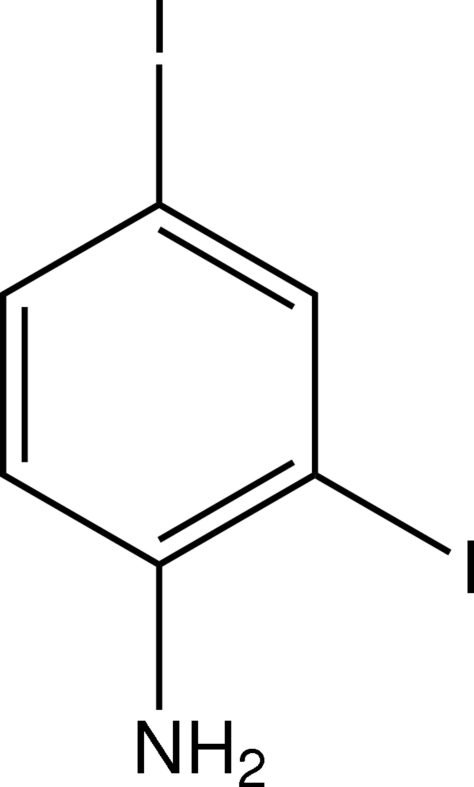


## Experimental

### 

#### Crystal data

C_6_H_5_I_2_N
*M* *_r_* = 344.91
Orthorhombic, 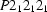

*a* = 4.3870 (1) Å
*b* = 10.9626 (3) Å
*c* = 16.9778 (4) Å
*V* = 816.51 (3) Å^3^
*Z* = 4
Mo *K*α radiation
μ = 7.62 mm^−1^
*T* = 200 K
0.30 × 0.18 × 0.18 mm

#### Data collection

Oxford Diffraction Gemini-S Ultra CCD-detector diffractometer
Absorption correction: multi-scan (**SADABS**; Sheldrick, 1996 ▶) *T*_min_ = 0.146, *T*_max_ = 0.250
6739 measured reflections
1873 independent reflections
1790 reflections with *I* > 2σ(*I*)
*R*_int_ = 0.024

#### Refinement

*R*[*F* ^2^ > 2σ(*F* ^2^)] = 0.018
*wR*(*F* ^2^) = 0.038
*S* = 1.05
1873 reflections
90 parameters
H atoms treated by a mixture of independent and constrained refinement
Δρ_max_ = 0.38 e Å^−3^
Δρ_min_ = −0.47 e Å^−3^
Absolute structure: Flack (1983 ▶), 737 Friedel pairs
Flack parameter: −0.03 (4)

### 

Data collection: *CrysAlis CCD* (Oxford Diffraction, 2008 ▶); cell refinement: *CrysAlis RED* (Oxford Diffraction, 2008 ▶); data reduction: *CrysAlis RED*; program(s) used to solve structure: *SHELXS97* (Sheldrick, 2008 ▶) within *WinGX* (Farrugia, 1999 ▶); program(s) used to refine structure: *SHELXL97* (Sheldrick, 2008 ▶) within *WinGX* (Farrugia, 1999 ▶); molecular graphics: *PLATON* (Spek, 2009 ▶); software used to prepare material for publication: *PLATON*.

## Supplementary Material

Crystal structure: contains datablocks global, I. DOI: 10.1107/S1600536809030438/is2440sup1.cif

Structure factors: contains datablocks I. DOI: 10.1107/S1600536809030438/is2440Isup2.hkl

Additional supplementary materials:  crystallographic information; 3D view; checkCIF report

## Figures and Tables

**Table 1:** Hydrogen-bond geometry (Å, °)

*D*—H⋯*A*	*D*—H	H⋯*A*	*D*⋯*A*	*D*—H⋯*A*
N1—H11⋯I2	0.77 (3)	2.81 (3)	3.283 (4)	122 (3)
N1—H12⋯N1^i^	0.80 (4)	2.30 (4)	3.106 (5)	180 (5)

Symmetry code: (i) 
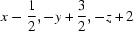
.

**Fig. 1.**
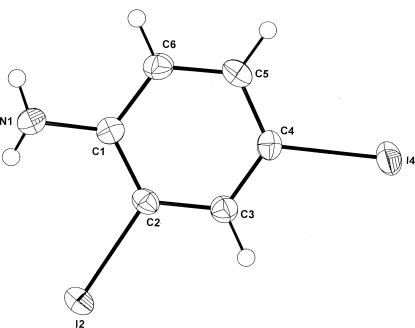
Molecular configuration and atom naming scheme for (I). Displacement ellipsoids are drawn at the 50% probability level.

**Fig. 2.**
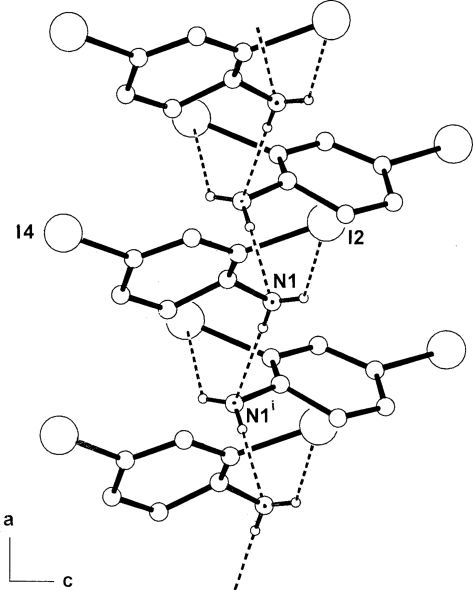
The one-dimensional hydrogen-bonded chain structure of (I) extending down the a axial direction of the unit cell, showing hydrogen-bonding associations as dashed lines. Non-interactive H atoms are omitted. [Symmetry code (i): x - 1/2, -y + 3/2, -z + 2].

## supplementary crystallographic information

### Comment

Although the crystal structures of a number of nitro-substituted iodoanilines including 3-nitro-2,4-diodoaniline have been reported (Garden *et al.*, 2002), that of the title compound 2,4-diiodoaniline C_6_H_6_I_2_N (I) has not been determined and the structure is reported here. The compound was isolated as the major crystalline product in the attempted synthesis of an adduct of 4,5-dichlorophthalic acid with 4-iodoaniline in aqueous ethanol. This conversion of 4-iodoaniline to 2,4-diiodoaniline has been reported previously (Dains *et al.*, 1935), where solid 4-iodoaniline was observed to undergo a *ca* 25% conversion to the diiodo analogue in a sealed container over a period of three years. Hodgson & Marsden (1937) also reported the ready formation of the diiodo derivative along with 4-iodoaniline from the reaction of aniline with iodine.

In the structure of (I) (Fig. 1), single weak intermolecular hydrogen bonds are found [N1—H1···N1^i^, 3.106 (5) Å; symmetry code: (i) *x* - 1/2, -*y* + 3/2, -*z* + 2] [graph set S(4) (Etter *et al.*, 1990)], linking the amine groups of 2_1_ screw-related molecules. These form one-dimensional chains which extend down the *a* cell direction in the unit cell (Fig. 2).

In this structure there are, not unexpectedly, short intramoleculer N—H···I interactions [N1···I2, 3.283 (4) Å], which are also present in the structure of 2,4-diiodo-3-nitroaniline [3.254 (7) Å (Garden *et al.*, 2002)]. However, unlike the nitro-derivative, no π–π stacking interactions are present in the structure of (I).

### Experimental

The title compound was formed in the attempted synthesis of a proton-transfer salt of 4,5-dichlorophthalic acid with 4-iodoaniline by heating together under reflux for 10 minutes 1 mmol quantities of the two reagents in 50 ml of 50% ethanol-water. After concentration to *ca* 30 ml, partial room temperature evaporation of the hot-filtered solution gave colourless needle prisms of 2,4-diiodoaniline [m.p. 368–389 K (O'Neil, 2001)] as the major product. This conversion of 4-iodoaniline to 2,4-diiodoaniline in the solid state has been reported previously (Dains *et al.*, 1935).

### Refinement

The hydrogen atoms of the amino group were located in a difference Fourier map and their positional and isotropic displacement parameters were refined freely. Other H-atoms were included in the refinement in calculated positions [C—H = 0.93 Å) and treated using a riding model approximation, with *U*_iso_(H) = 1.2*U*_eq_(C).

### Crystal data

**Table:**

C_6_H_5_I_2_N	*D*_x_ = 2.806 Mg m^−^^3^
*M**_r_* = 344.91	Melting point = 368–369 K
Orthorhombic, *P*2_1_2_1_2_1_	Mo *K*α radiation, λ = 0.71073 Å
Hall symbol: P 2ac 2ab	Cell parameters from 5620 reflections
*a* = 4.3870 (1) Å	θ = 3.0–32.2°
*b* = 10.9626 (3) Å	µ = 7.62 mm^−^^1^
*c* = 16.9778 (4) Å	*T* = 200 K
*V* = 816.51 (3) Å^3^	Needle, colourless
*Z* = 4	0.30 × 0.18 × 0.18 mm
*F*(000) = 616	

### Data collection

**Table:**

Oxford Diffraction Gemini-S Ultra CCD-detector diffractometer	1873 independent reflections
Radiation source: Enhance (Mo) X-ray tube	1790 reflections with *I* > 2σ(*I*)
graphite	*R*_int_ = 0.024
ω scans	θ_max_ = 27.5°, θ_min_ = 3.0°
Absorption correction: multi-scan (*SADABS*; Sheldrick, 1996)	*h* = −5→5
*T*_min_ = 0.146, *T*_max_ = 0.250	*k* = −13→14
6739 measured reflections	*l* = −22→18

### Refinement

**Table:**

Refinement on *F*^2^	Secondary atom site location: difference Fourier map
Least-squares matrix: full	Hydrogen site location: inferred from neighbouring sites
*R*[*F*^2^ > 2σ(*F*^2^)] = 0.018	H atoms treated by a mixture of independent and constrained refinement
*wR*(*F*^2^) = 0.038	*w* = 1/[σ^2^(*F*_o_^2^) + (0.0207*P*)^2^] where *P* = (*F*_o_^2^ + 2*F*_c_^2^)/3
*S* = 1.05	(Δ/σ)_max_ = 0.003
1873 reflections	Δρ_max_ = 0.38 e Å^−^^3^
90 parameters	Δρ_min_ = −0.47 e Å^−^^3^
0 restraints	Absolute structure: Flack (1983), 737 Friedel pairs
Primary atom site location: structure-invariant direct methods	Flack parameter: −0.03 (4)

### Special details

**Table:**

Geometry. Bond distances, angles *etc*. have been calculated using the rounded fractional coordinates. All su's are estimated from the variances of the (full) variance-covariance matrix. The cell e.s.d.'s are taken into account in the estimation of distances, angles and torsion angles
Refinement. Refinement of *F*^2^ against ALL reflections. The weighted *R*-factor *wR* and goodness of fit *S* are based on *F*^2^, conventional *R*-factors *R* are based on *F*, with *F* set to zero for negative *F*^2^. The threshold expression of *F*^2^ > σ(*F*^2^) is used only for calculating *R*-factors(gt) *etc*. and is not relevant to the choice of reflections for refinement. *R*-factors based on *F*^2^ are statistically about twice as large as those based on *F*, and *R*- factors based on ALL data will be even larger.

### Fractional atomic coordinates and isotropic or equivalent isotropic displacement parameters (Å^2^)

**Table:**

	*x*	*y*	*z*	*U*_iso_*/*U*_eq_	
I2	0.57888 (5)	0.42657 (2)	1.08868 (1)	0.0293 (1)	
I4	0.48489 (5)	0.28546 (2)	0.75021 (1)	0.0330 (1)	
N1	0.1721 (9)	0.6552 (3)	1.0212 (2)	0.0291 (11)	
C1	0.2299 (7)	0.5690 (3)	0.9630 (2)	0.0218 (9)	
C2	0.4096 (8)	0.4658 (3)	0.97570 (19)	0.0221 (9)	
C3	0.4807 (8)	0.3859 (3)	0.9156 (2)	0.0247 (9)	
C4	0.3689 (8)	0.4074 (3)	0.8407 (2)	0.0235 (10)	
C5	0.1876 (8)	0.5075 (3)	0.8256 (2)	0.0260 (11)	
C6	0.1216 (9)	0.5877 (3)	0.8865 (2)	0.0278 (11)	
H3	0.60280	0.31820	0.92530	0.0300*	
H5	0.11070	0.52100	0.77530	0.0310*	
H6	0.00190	0.65580	0.87610	0.0330*	
H11	0.190 (8)	0.626 (3)	1.062 (2)	0.038 (9)*	
H12	0.043 (8)	0.704 (4)	1.010 (2)	0.040 (9)*	

### Atomic displacement parameters (Å^2^)

**Table:**

	*U*^11^	*U*^22^	*U*^33^	*U*^12^	*U*^13^	*U*^23^
I2	0.0335 (1)	0.0341 (1)	0.0202 (1)	0.0009 (1)	−0.0038 (1)	0.0021 (1)
I4	0.0383 (1)	0.0389 (1)	0.0219 (1)	0.0022 (1)	0.0017 (1)	−0.0069 (1)
N1	0.037 (2)	0.0222 (16)	0.028 (2)	0.0055 (15)	−0.0021 (16)	0.0013 (15)
C1	0.0214 (15)	0.0191 (16)	0.0248 (18)	−0.0039 (15)	0.0014 (14)	0.0021 (14)
C2	0.0251 (16)	0.0226 (16)	0.0186 (17)	−0.0034 (15)	−0.0009 (14)	0.0032 (11)
C3	0.0289 (18)	0.0218 (14)	0.0234 (17)	−0.0007 (11)	−0.0010 (16)	0.0015 (12)
C4	0.0252 (17)	0.0226 (18)	0.0228 (18)	−0.0031 (13)	0.0028 (14)	−0.0029 (13)
C5	0.027 (2)	0.0313 (19)	0.0197 (19)	−0.0028 (14)	−0.0018 (15)	0.0044 (14)
C6	0.0323 (19)	0.0236 (18)	0.0276 (19)	0.0028 (15)	0.0007 (15)	0.0051 (13)

### Geometric parameters (Å, °)

**Table:**

I2—C2	2.101 (3)	C2—C3	1.381 (5)
I4—C4	2.099 (3)	C3—C4	1.383 (5)
N1—C1	1.391 (5)	C4—C5	1.379 (5)
N1—H12	0.80 (4)	C5—C6	1.388 (5)
N1—H11	0.77 (3)	C3—H3	0.9300
C1—C6	1.398 (5)	C5—H5	0.9300
C1—C2	1.396 (5)	C6—H6	0.9300
			
H11—N1—H12	124 (4)	I4—C4—C3	118.6 (2)
C1—N1—H11	110 (3)	C3—C4—C5	120.7 (3)
C1—N1—H12	114 (3)	C4—C5—C6	119.1 (3)
N1—C1—C6	119.9 (3)	C1—C6—C5	121.9 (3)
N1—C1—C2	123.0 (3)	C2—C3—H3	120.00
C2—C1—C6	117.0 (3)	C4—C3—H3	120.00
I2—C2—C1	120.4 (2)	C4—C5—H5	120.00
I2—C2—C3	117.7 (2)	C6—C5—H5	121.00
C1—C2—C3	121.9 (3)	C1—C6—H6	119.00
C2—C3—C4	119.4 (3)	C5—C6—H6	119.00
I4—C4—C5	120.7 (2)		
			
N1—C1—C2—I2	5.0 (5)	C1—C2—C3—C4	−0.7 (5)
N1—C1—C2—C3	−175.5 (3)	C2—C3—C4—I4	179.3 (3)
C6—C1—C2—I2	−178.9 (2)	C2—C3—C4—C5	0.0 (5)
C6—C1—C2—C3	0.6 (5)	I4—C4—C5—C6	−178.5 (3)
N1—C1—C6—C5	176.5 (3)	C3—C4—C5—C6	0.9 (5)
C2—C1—C6—C5	0.3 (5)	C4—C5—C6—C1	−1.0 (5)
I2—C2—C3—C4	178.8 (3)		

### Hydrogen-bond geometry (Å, °)

**Table:**

*D*—H···*A*	*D*—H	H···*A*	*D*···*A*	*D*—H···*A*
N1—H11···I2	0.77 (3)	2.81 (3)	3.283 (4)	122 (3)
N1—H12···N1^i^	0.80 (4)	2.30 (4)	3.106 (5)	180 (5)

Symmetry codes: (i) *x*−1/2, −*y*+3/2, −*z*+2.
